# When should researchers cite study differences in response to a failure to replicate?

**DOI:** 10.1007/s10539-022-09873-y

**Published:** 2022-09-02

**Authors:** David Colaço, John Bickle, Bradley Walters

**Affiliations:** 1 Munich Center for Mathematical Philosophy, LMU Munich, Munich, Germany; 2 Department of Philosophy and Religion, Mississippi State University, Starkville, USA; 3 Department of Neurobiology and Anatomical Sciences, University of Mississippi Medical Center, Jackson, USA; 4 Department of Otolaryngology and Communicative Sciences, University of Mississippi Medical Center, Jackson, USA; 5 Department of Advanced Biomedical Education, University of Mississippi Medical Center, Jackson, USA

**Keywords:** Replication, Translation, Model organisms, Experiment, Neuroscience

## Abstract

Scientists often respond to failures to replicate by citing differences between the experimental components of an original study and those of its attempted replication. In this paper, we investigate these purported *mismatch explanations*. We assess a body of failures to replicate in neuroscience studies on spinal cord injury. We argue that a defensible mismatch explanation is one where (1) a mismatch of components is a *difference maker* for a mismatch of outcomes, and (2) the components are *relevantly different* in the follow-up study, given the scope of the original study. With this account, we argue that not all differences between studies are meaningful, even if they are difference makers. As our examples show, focusing only on these differences results in disregarding the representativeness of the original experiment’s components and the scope of its outcomes, undercutting other epistemic aims, such as translation, in the process.

## Introduction

A “crisis” in medicine (Ioannidis 2005) and psychology (Open Science Collaboration 2015) has brought with it an increased scrutiny of the role of replication in science. This scrutiny includes debates about the relative importance of replication over other epistemic practices (Feest 2019), the social mechanisms that promote replication (Romero 2018), and even the meaning of ‘replication’ itself (Machery 2020).

Failures to achieve epistemic aims fuel worries about replication in scientific fields beyond medicine and psychology. For instance, a program called “Facilities of Research Excellence—Spinal Cord Injury” (FORE-SCI), established by the National Institute of Neurological Disorders and Stroke (NINDS), began after perceived failures to translate results in animal studies of spinal cord injury to therapeutic interventions in human patients. Researchers designed FORE-SCI to replicate several notable spinal cord injury studies (Steward et al. 2012a). This program ended up with a “surprising preponderance of failures to replicate,” and researchers proposed 11 “reasons for a failure to replicate” (Steward et al. 2012a, p. 601).

While these neuroscientists clearly value replication, their 11 reasons for these failures mostly address differences between the experimental components of the original study and its attempted replication. While concerns over the requisite degree of “exactness” or “directness” for replications are common in recent literature (Schmidt 2009; Zwaan et al. 2018; Feest 2019; Romero 2019), and study differences are often noted by practicing scientists, there remains a question that has received little explicit discussion: under what circumstances should researchers rest content with a *mismatch explanation* for reported failures to replicate?

In this paper, we account for mismatch explanations. A mismatch explanation is one where (1) the match of experimental components is a difference maker for the match of experimental outcomes, and (2) the components are relevantly different in the follow-up study, given the scope of the original study. With our account, we argue that not all differences between studies are candidates for mismatch explanations, even if they are difference makers. As examples from the FORE-SCI program show, addressing study differences without consideration of the original study’s scope can undercut other epistemic aims, such as translation. Thus, our aim in this paper is not to deny that mismatch explanations are ever defensible. We think that they are. Rather, we question whether cases like some of those reflected in FORE-SCI meet both conditions for defensible mismatch explanations.

In “Section Mismatch explanations”, we give an account of mismatch explanations. In “Section Attempted spinal cord injury replications”, we discuss the FORE-SCI research program, in which scientists cite study differences in response to several reported replication failures in spinal cord injury research. In “Section Assessing a purported mismatch explanation”, we discuss a FORE-SCI case in more detail and assess whether a mismatch explanation is appropriate. In Section “Why some study differences don’t matter for replication”, we address the consequences of explaining replication failures solely in terms of difference makers, including the effects of these explanations on other epistemic aims. We also propose a method for resolving inappropriate appeals to mismatch explanations in the future. We conclude by speculating that mismatch explanations are popular for several reasons, including researchers’ concerns about culpability.

## Mismatch explanations

The term ‘replication’ is used in a variety of ways within and between scientific disciplines. In some cases, it is synonymous with reproducing an earlier outcome, and it is often equated to producing robust supportive evidence or extrapolating scientific results (Schmidt 2009). Debates about what we want from a replication also are common. These debates result in part from the fact that studies will invariably differ in some manner (Feest 2019; Romero 2019). Thus, there will always be differences between an original study and its follow-up.

Given that differences between studies are inevitable, when is it appropriate to explain reported replication failures in terms of these differences? In answering this question, we do not identify some novel kind of explanation. Instead, we account for when a mismatch serves as an appropriate probe into whether a follow-up study counts as a bona fide replication attempt and thus has epistemic consequences. We present two conditions for a defensible mismatch explanation. The first condition is that any cited difference between two studies’ components must be a reason the two studies have different outcomes. We call this the *difference maker condition*. Whether a mismatch meets this condition is determined by manipulating the match of the components and measuring the match of the outcomes. For this reason, our account satisfies an interventionist account of causal explanation (Woodward 2005). This condition captures the fact that differences alone are not enough. They must be difference makers.

The difference maker condition alone does not capture everything of importance for a mismatch explanation. To understand why, we address the nature of experimental components and outcomes of a study. First, how do we construe experimental components and their *representativeness*? To match an original study in a replication, we must hold some components constant. These are what Machery’s resampling account of replication calls *fixed factors* (2020). Take a study that tests the specific effect of ibuprofen on symptom severity when used in COVID-19 patients. If this drug is substituted with acetaminophen in a follow-up trial, this new study does not follow the original. The two drugs and how they interact with the SARS-CoV-2 virus and its receptors differ (Gonzalez-Paz et al. 2020). By contrast, there are what the resampling account calls *random factors*. Take a study that tests the effect of opioids for pain management in cancer patients. As the mechanism of action across several different opioid medications is expected to be similar for pain relief, the test of any single opioid medication might be considered representative of other opioids. In this case, replicators need not use (say) oxycodone if it was used in the original study. In this study, oxycodone stands for the population of opioids. However, the replicators cannot substitute just *any* drug in the follow-up trial. They must use an opioid. Random factors need not be held constant but must be chosen from some population to which they are representative. The character of this population is dependent upon the scope of the experimental outcome and stated conclusions. In other words, the original researchers' intended scope of their findings should inform which populations are representative for random factors. Replicators must match a factor’s representativeness when attempting to replicate a study.^1^

Second, how do we construe experimental outcomes and their *scope*? In how general of terms are the scientific outcomes to be described by researchers (Simons et al. 2017; Nosek and Errington 2020)? Some degree of generality beyond the specifics of an individual experiment is expected if outcomes apply to something more general than the sample of individuals or specific conditions studied in any single experiment. For instance, rodent research that is intended to translate to therapeutic interventions in humans must have outcomes whose scope applies to humans, or at least to more than just the cohort of mice or rats tested in an individual study. The scope of these outcomes relates to the representativeness of the components.

Considering representativeness and scope, the second condition for a mismatch explanation is that a difference must count as a relevant mismatch. We call this the *relevance condition*. When are mismatches relevant? First, fixed factors can be mismatched. For instance, the original researchers or replicators might inadvertently introduce a component into their study that results in a confound, or a factor that is distinct from, but covaries with, the hypothesized effect (Schickore 2019).^2^ Second, random factors can be mismatched. This occurs when a component in the follow-up is not representative of random factors in the original study, given the original study’s scope. This condition captures that not all differences matter for replication, even if they are difference makers. Returning to the opioid example, using hydrocodone instead of oxycodone is not a relevant mismatch if oxycodone was intended to be representative of any opioid. Using a drug that is not an opioid in this example, however, is not representative, so this would be a relevant mismatch.^3^

For the purposes of this paper, we follow the resampling account and take replication to establish *reliability*: when fixed factors are held constant, testing representative random factors should result in the same outcome with a high frequency (Machery 2020). If fixed factors are not held constant or the random factors of the follow-up are not representative, then the follow-up is not a replication attempt. This requirement of the resampling account captures one reason mismatch explanations can be valuable. A follow-up study cannot satisfy both the relevance condition and the resampling account. Returning to the example, either hydrocodone is representative of the opioid population and its use in a follow-up should count as a replication attempt, or it is a relevant difference, and its use in a follow-up study should not count as a replication attempt. A mismatch explanation that meets the relevance condition entails that the follow-up study does not count as a replication failure. More importantly, a mismatch explanation pinpoints a plausible cause for the difference in outcomes that does not call the reliability of the original study into question.

## Attempted spinal cord injury replications

Given the debilitating consequences of human spinal cord injuries, researchers induce spinal trauma in animal models to learn how to “reduce secondary injury, improve recovery, or enhance axon regeneration” in hopes that they can extend this knowledge to humans (Steward et al. 2012a, p. 597). In 2003, NINDS developed a program, FORE-SCI, to promote and fund replications of several notable studies in this area of research. FORE-SCI was motivated by a perceived lack of therapeutic interventions in humans, despite “apparent progress,” or reports of successful original studies in animal models (Steward et al. 2012a, p. 597). This lack of translation also raised concerns about the reliability of this research that was rarely, if ever, replicated prior to FORE-SCI.

In hindsight, these concerns were well-founded. In FORE-SCI, there were a “surprising preponderance of failures to replicate” spinal cord injury studies (Steward et al. 2012a, p. 601). Of 12 reported replication attempts from FORE-SCI, six were “no replication,” two were “partial replication,” one was “mixed results,” one was inconclusive, and two were successful (Steward et al. 2012a, p. 600). If these outcomes were not troubling enough, one successful replication resulted in a lower effect compared to the original study, and the other was only “replicated” following a discovery of a mismatch resulting from a previously unqualified component of the original study (more on this example in Sect. 4). While not a systematic attempt to replicate studies in a field (unlike, e.g., Open Science Collaboration 2015), these outcomes are troubling for spinal cord injury research. On an optimistic interpretation, half of the chosen studies failed to replicate. On a pessimistic interpretation, *all* attempts involved some complications in replicating the original study.

Closer inspection of one failure to replicate from FORE-SCI is insightful. Researchers set out to replicate a study designed to test the effect of inosine on transmidline axons in the rat corticospinal tract (Benowitz et al. 1999). Inosine is a purine nucleoside, which can affect a neuron’s axonal growth in vitro. In the original study, inosine injection was reported to cause pyramidal cells to grow and sprout their axons into denervated spinal white matter, suggesting that inosine intervention may be an effective means to “restore essential circuitry after injury to the central nervous system” (Benowitz et al. 1999, p. 13486). In other words, this study putatively showed that a natural metabolite could reverse some of the damage caused by a spinal cord injury by supplanting axonal formations and reestablishing connections in a spinal cord. However, Steward and colleagues, with the backing of FORE-SCI, did not measure any effect of this sort, reporting: “inosine-treated rats did not exhibit the transmidline sprouting reported in the original study” (2012b, p. 663). This was despite their best efforts to match the components of the original study.

While they note that “there are no technical explanations for [their] failure to detect the growth reported by Benowitz et al.,” Steward and colleagues still go on to consider some of the “technical details” that the original and replication studies might not share (2012b, p. 672). First, Steward and colleagues cite differences between the rats used in the original study and replication. Despite acquiring the same strain from the same vendor, they propose that genetic drift may have led to changes during the 12 years between the respective studies. Second, the replicators suggest that rat rearing and preparation were possibly different. Third, the replicators could not rule out potential differences between the lesions, despite their attempts to match the methods by which they induced these lesions. Fourth, differences between how the axonal formations were traced and labeled between the respective studies were cited, though Steward and colleagues suggest that there is no obvious reason the mismatch of outcomes should depend on these differences.

Benowitz, the principal researcher of the original study, responded to this reported replication failure. He notes that “the study by Steward et al. is done carefully but differs from the original study in two ways that may have contributed substantially to the divergent result” (Benowitz 2012, p. 674). These differences are mentioned above: rearing conditions and labeling technique. Benowitz also suggests that how the midline was determined in the rats and the data analysis might have contributed to the failure to replicate as well. While Benowitz suggests these differences may explain the reported replication failure, he provides no evidence in defense of this claim in his response.

This consideration of differences is representative of the reasons presented in the concluding FORE-SCI report: overall, 11 reasons for failures to replicate are stated (Steward et al. 2012a, p. 601). These include:

1. Type-1 errors
2. Differences in experimental details between original study and replication
3. Greater variability in the replication as compared to original study
4. Mistakes by replicators who were unskilled in using injury models or methods
5. Differences in animal models between original study and replication
6. Differences in lesions between original study and replication
7. Differences in care for animals between original study and replication
8. Differences in reagents between original study and replication
9. Bias
10. A non-robust effect
11. Scientific misconduct

Thus, while statistical (and ethical) reasons appear, this list highlights a much greater focus on differences between the experimental components of original studies and replication attempts.

While we focus on FORE-SCI in this paper, researchers’ tendency to cite study differences in efforts to explain a failure to replicate can be found in other areas of cognitive science and neuroscience as well. When skeptics (Karin-D'Arcy and Povinelli 2002) discuss difficulties in replicating animal mindreading studies that deploy competitive feeding paradigms (Hare et al. 2001), Heyes claims that “these difficulties suggest that the published reports… have not contained sufficient detail to allow their methods to be reproduced in other laboratories” (2015, p. 316; see also Halina 2021). In the history of neuroscience, failures to replicate undermined claims that memories can be transferred. In response, proponents claimed that all failures to replicate were due to differences between original and follow-up studies (Colaço 2018, p. 36). In psychology, the field wherein the bulk of recent replication debates have taken place, study population differences routinely are cited as explanations for reported replication failures, though they may be met with skepticism (Owens 2018). Together, these cases support the claims that the “reaction of some scientists whose work fails to replicate is to emphasize that the replication attempts introduce substantive variations that explain the failures” (Romero 2019, p. 5) and “if a direct replication fails to obtain the same result as the original study, researchers may question… whether there is a misunderstanding about the understanding of the essential features required to produce an effect” (Zwaan et al. 2018, p. 4). These examples supply evidence that the FORE-SCI cases are not anomalies, and we invite readers to consider whether scientists attempt to explain replication failures in terms of study differences in their field.

Invoking differences in these kinds of cases also resembles, at least rhetorically, responses to other alleged scientific failures that philosophers of science address. For instance, many of the responses in FORE-SCI are akin to Duhem’s claims about underdetermination. Just as a disconfirmation or failed prediction leaves open the opportunity for researchers to blame an auxiliary assumption rather than their main hypothesis (Ariew 1984), FORE-SCI’s troubling results leave open the opportunity for researchers to blame the differences between original and follow-up studies rather than anything to do with the reliability of the original study (or for that matter the ethical character of its researchers).

## Assessing a purported mismatch explanation

The FORE-SCI example and accompanying discussion in Sect. 3 show that researchers often cite differences between experimental components in their attempts to explain reported replication failures. This is problematic, as no replication can be exact.^4^ It also meets neither of our conditions for a mismatch explanation. There is no reason for us to think these differences are difference makers, nor is there consideration of whether they are relevant. However, some FORE-SCI examples include a search for difference makers. We analyze one of these cases, which resulted in a “successful replication.”

### When researchers search for differences

A group of FORE-SCI researchers set out to replicate a study designed to test the role of sulfonylurea receptor 1-regulated (SUR1-regulated) cation channels in progressive hemorrhagic necrosis, or PHN (Simard et al. 2007). This form of necrosis, resulting from acute spinal injury, leads to “devastating loss of spinal cord tissue, cystic cavitation of the cord, and debilitating neurological dysfunction” (Simard et al. 2007, p. 2105). The original study reports that the activity of SUR1-regulated channels can be controlled via the inhibition of SUR1 by the drug glibenclamide (Simard et al. 2007, p. 2109; Popovich et al. 2012, p. 615). When this inhibitor was tested, necrosis and its consequences were reduced. As the original researchers put it, the “block of SUR1 is associated with significant improvements in all of the characteristic manifestations of PHN” (Simard et al. 2007, p. 2111). Based on these findings, the original researchers claim that SUR1-regulated channels are a suitable target for spinal cord injury therapy and that glibenclamide is “especially attractive for translational use in human SCI [spinal cord injury]” (Simard et al. 2007, p. 2112).

**Fig. 1:**
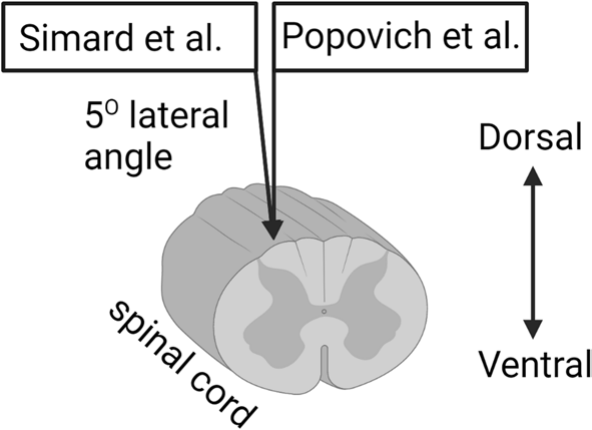
Visualization of injury induction in Simard et al. (2007) versus Popovich et al. (2012) (image generated via biorender.com)

Popovich and colleagues set out to replicate Simard and colleagues’ study “in which glibenclamide was found to limit PHN in a model of rat cervical contusion injury” (Popovich et al. 2012, p. 615). They were able to do so in the end: like Simard and colleagues, they determined that glibenclamide can limit PHN. However, Popovich and colleagues initially reported a failure to replicate, as there were no “differences in the magnitude of intraspinal hemorrhage between” experimental and control groups (2012, p. 619). Only after “extensive discussions” with the principal researchers of the original study did the two teams discover a potential mismatch: Popovich and colleagues induced contusion injuries in a manner that was dissimilar to the original study. The original study induced the injury at about a 5° angle deviation from the dorsoventral axis of the spinal cord (see Fig. 1), while the initial replication attempt induced the injury parallel to the dorsoventral axis (Popovich et al. 2012, p. 619). Though several factors suggest they cause the same injury, these two methods differently induce contralateral damage to the spinal cord. Further, the original method proved difficult to execute because it departs from more standardized means of inducing spinal cord injury. Following this discovery, the research teams from the original study and replication worked together to match the method in subsequent replication attempts.

Simard and Gerzanich, who performed the original study and informed the later replication attempts, responded to Popovich and colleagues’ report. They note that their description of methods in their original study (Simard et al. 2007) insufficiently detailed the injury induction, which explains why Popovich and colleagues did not match this method until after their discussion (Simard and Gerzanich 2012). However, this was not viewed as an omission in the original report. In their response, Simard and Gerzanich note: “we simply assumed that any unilateral injury would produce this result. We didn't realize that some unilateral injuries could lead to immediate bilateral hemorrhage that would mask contralateral spread of hemorrhage” (2012, p. 623). They go on to ask whether “the requirement for an identical injury in the replication experiment [implies] that other injuries would not be benefited by treatment with glibenclamide,” to which they answer that “it depends on the question being asked, and the outcome being measured” (Simard and Gerzanich 2012, p. 623). With this irresolute answer, they list several caveats to the scope of their original outcome.

### Analyzing this case

Certainly, there is a difference between the injury method in the original study and the initial attempt to replicate it. However, does it meet both conditions for a mismatch explanation? In this case, researchers tested whether the change in injury method varied with the change in outcome. When the same injury method was used, the outcome was matched in a later follow-up. In this follow-up, the replicators revised how their replication attempts were performed, which required a significant degree of experimental expertise that came from the original researchers. Determining the difference in injury method was neither a speculation nor an ad-hoc excuse, and it turned out to be a difference maker. Thus, the difference maker condition is satisfied.

What about the relevance condition? The report of the original study lacks detail on this study’s scope and the representativeness of its components. Given the translational aspirations of the original study, we can speculate that the intervention and treatment should represent human clinical cases where spinal cord injuries occur at a variety of angles (Smith et al., 2005). This vagueness is evidence for the concern that scientists often do not clarify whether experimental components are fixed or random factors (Machery 2020, p. 561). However, speculation is not necessary, as Simard and Gerzanich admit that they assumed that any unilateral injury would result in the same outcome. Likewise, in the report of the original study, they make broad statements about the scope of their findings, stating that the “block of SUR1 is associated with significant improvements in *all* of the characteristic manifestations of PHN” (Simard et al. 2007, p. 2111, emphasis added). They do not specify that their results might only apply to subtypes of spinal cord injuries, such as unilateral, cervical, or of only a specific impact angle. Given these concluding statements, one could argue that any method of spinal cord injury that induces PHN should be considered part of the population that is representative based on the stated, intended scope. A spinal cord injury that deviated only by 5° but maintained similar level (cervical) and impact energy is well within the bounds of being representative. Furthermore, Popovich and colleagues used a unilateral injury method, which is representative of the population of injury methods akin to the original study. Thus, this case does not satisfy the relevance condition. Consistent with Machery’s resampling account, the injury method is not a fixed factor. Simard and colleagues were not studying whether PHN caused by the specific injury they induced can be treated with glibenclamide. Rather, and based on their own comments, the injury method is a random factor, one chosen by the original researchers from a population of methods as representative of any unilateral injury. On our account, the original follow-up is a failure to replicate, and a mismatch explanation is not appropriate in this case.

It was only after the original researchers and replicators determined that the injury method is a difference maker that they suggested it should be treated as a fixed rather than random factor. This fact highlights that their discovery of a difference maker led both research groups to explain the initial failure in terms of the match of the injury method. When this injury method was later matched, they called the case a successful replication. At the same time, the relevance condition has not been prioritized. Even though the responses from the original researchers and the replicators suggest that they both assumed that the findings would generalize to a variety of spinal cord injuries, this fact is not considered at all when they claim to successfully replicate the original study in the end. Thus, this case provides an example of how difference makers can be prioritized over the relevance of a potential mismatch.

## Why some study differences don’t matter for replication

In Sect. 2, we presented two conditions for when a mismatch serves as an appropriate probe into whether a follow-up study counts as a bona fide replication attempt: the difference maker condition and the relevance condition. The case in Sect. 4 satisfies only the difference maker condition. For this reason, we argue that it is not a defensible mismatch explanation. It is an example of why discovering a difference maker alone does not entail that a mismatch explanation is appropriate.

### The importance of the relevance condition

At this point, the reader might object to the relevance condition. After all, the researchers in this case identified a component difference between the original study and the follow-up that is demonstrably a difference maker for their respective outcomes. Beyond quibbles about what really counts as a ‘mismatch,’ this case resulted in an explanation of the mismatch of outcomes that is not due to the unreliability of the original study. Even if there was some vagueness initially about the representativeness of the original injury method, why should this case not be explained in terms of the identified mismatch?

This objection is consistent with FORE-SCI researcher responses. In Sect. 4, a concern about the exact way the spinal cord injury is induced in rats was the response of the original researchers. This focus on differences also is illustrated by Benowitz’s reaction to the failure to replicate, discussed in Sect. 3. It further is reflected in the 11 reasons supplied above; six of them directly cite study differences. One may try to explain this ratio by arguing that within a single experimental approach, differences may be myriad, but there is only one way that replicators could attribute replication failure to a false positive, or similarly perhaps only one way to accuse someone of fraud. However, the list includes general differences, such as the difference between experimental details expressed in (2), while at the same time, the list cites specifics in (5), (6), (7), and (8). Even though (2) encompasses (5) through (8), the authors nonetheless included these as separate reasons. Similarly, the replicators could have devised several specific ways in which the methods or results could be manipulated, but they offered only (11) to encompass all the actions that could be categorized as misconduct.

While this objection may seem appealing, it reveals serious concerns with an endeavor to explain all reported replication failures solely in terms of difference makers. In this case, the original study can be counted as having a successful replication if one more narrowly construes the scope of the original’s outcomes after the fact. This amounts to a post-hoc reappraisal of Simard and colleagues’ conclusions only applying to a subtype of spinal cord injury rather than all injuries. This comparatively narrow outcome is not consistent with what the original researchers, by their own admission, assumed. Only after the initial failure to replicate and the discovery of a difference maker did the original researchers recognize that the injury method is not representative of all unilateral injuries.

At first glance, this situation might seem unproblematic. In fact, the reader might be sympathetic to researchers’ interests in uncovering difference makers and better controlling for them, further supporting this objection. These are admirable practices, but we argue that their application to replication comes at a cost. This cost is material: researchers must spend time, money, and resources hunting down potential difference makers. In the FORE-SCI studies, including the two we have detailed, many differences were pursued (Steward et al. 2012a, p. 600), but only one difference maker was discovered amongst 12 replication attempts. Thus, despite the time, money, and resources spent, the researchers’ endeavors to hunt down difference makers largely did not help them in explaining the preponderance of failures to replicate in FORE-SCI.

More importantly, this cost is also epistemic. If researchers care only about difference makers, they open the opportunity to undermine other epistemic aims, such as translation. This is ironic, as the motivating concern for FORE-SCI was to determine why “not one of the many strategies reported as having promising effects were translated into successful therapies” in humans (Steward et al. 2012a, p. 597).

Why might translation suffer when researchers focus exclusively on difference makers, regardless of whether they satisfy the relevance condition? Translatability is the epistemic aim of extending study outcomes to clinical contexts. For FORE-SCI, the aim is to extend outcomes on treatment of spinal cord injuries in rodent model organisms to human patients who have these injuries. For this translation to be successful, the outcomes of the study must have a scope that extends to these clinical contexts. Correspondingly, the components of the study must be representative of the kinds of clinical conditions through which human spinal cords are damaged. The treatment must be effective on spinal cord injuries, rather than merely on one kind of spinal cord injury in rats. If one disregards the relevance condition and merely narrows representativeness and scope in the face of a difference maker, one thus ignores what clinical contexts one’s research can translate to and possibly might undercut any translational aim one has.

These consequences of disregarding the relevance condition can be shown with the FORE-SCI case. Initially, researchers claimed that an intervention reduced, remediated, or resolved spinal cord injury. However, researchers cannot make claims of such generality after focusing on difference makers and disregarding the relevance condition. Many components that were initially taken to be broadly representative random factors now must be reconstrued. Thus, researchers can no longer claim that “the drug improves a spinal cord injury symptom.” Instead, they can only say that “the drug improves a symptom *of a very particular kind of spinal cord injury in organisms that are of a specific species and strain, have been manipulated in a very particular way, and reared in a specific colony, *etc*.*” While the first claim shows promise for translating to the clinic, the latter raises many questions. Do humans exhibit these symptoms in the same manner and chronology? Do humans receive spinal cord injuries like the kind induced in the original study? Are any of the specified rodent rearing conditions akin to human development?

This is not to say that the answers to these questions are necessarily “no,” or that the research entirely lacks translational value. Translation is distinct from the epistemic aim of inductive generalization, as translation can be achieved even if the study’s outcomes do not apply to diverse kinds of clinical contexts. For instance, humans suffer common but characteristic brain injuries, such as when an unbuckled driver predictably sustains a frontal injury after hitting the steering wheel with their forehead during a car accident. Studies using an injury induction method that is representative of this specific, real-world scenario might translate into therapies for humans with this very specific, but also quite common, injury, even if they do not generalize to all, or any other, types of brain injuries. This feature of translation is akin to the epistemic aim of extrapolation, which is best motivated by anatomical or mechanistic similarities between the model and target systems (Steel 2007).

Nonetheless, for the case from FORE-SCI that we recounted in Sect. 4, translation will be much more limited than the authors of the original report stated or implied. Given that translation concerns motivated FORE-SCI, and there is no reason to think that a 5° angle deviation from midline in cervical injury in rats represents some clinically important factor of human spinal cord injuries independently of it being a difference maker, researchers likely will not promote translation if they disregard the relevance condition (short of a major and surprising discovery). More seriously, disregarding the relevance condition might result in researchers not addressing the clinical extension of their study outcomes. They may focus only on the difference makers themselves, rather than what these difference makers represent in the clinical contexts to which they want their research to translate.

Do we think researchers should never investigate difference makers? No; learning about difference makers is undoubtedly important in the generation of scientific knowledge. However, we are skeptical of explaining all reported replication failures in terms of difference makers without considering the relevance condition. If one merely takes any difference maker to explain differences between studies’ outcomes, we are no longer talking about the replication of studies that have scopes beyond the literal components of the studies and their interactions. There is no assessment of the reliability of a study with a certain scope if that scope is so malleable post hoc, and we argue that this is a significant loss to how we should understand not only reliability but also other epistemic aims like translation, generalization, and robustness. Even if researchers gain scientific knowledge about these difference makers, they give up on the extension of these difference makers to diverse experimental contexts, let alone clinical ones. Thus, we do not deny that difference makers matter, but we think that they do not always matter for replication.

Our distinction between the general scientific value of difference makers and their importance in diagnosing replication attempts answers a question the reader might have about the strength of our thesis. That seemingly similar injury induction methods result in different outcomes might interest researchers. For this reason, they may ask whether this difference maker reflects something important for the study of spinal cord injuries.^5^ If the difference maker indeed reflects something important, it could be consequential for their epistemic aims, including translation. We do not deny this possibility. If researchers wish to investigate how two (or more) injury induction methods can result in different outcomes, they should do so. They might discover something interesting about these methods in the process. What they should not do, however, is take this difference maker alone to resolve a reported replication failure.

There are other reasons to be skeptical of explaining all replication failures in terms of difference makers. A focus on differences limits discussion of statistical standards, of combating questionable research practices, revising how methods are reported, or more precisely reporting the scope of conclusions. Likewise, focusing on differences assumes that the original study was correct, which illustrates “a tacit assumption…that the original study is a flawless, expertly performed piece of research that reported a true effect” (Zwaan et al. 2018, p. 6). Indeed, as we saw here, this focus is perhaps exacerbated when testing differences leads to the discovery of a difference maker. In such cases, the original studies persist in some form, albeit with an outcome of much narrower scope, leading to a pseudo-resolution that allows researchers to downplay any issues beyond the difference. It is here we see that invoking mismatch explanations is akin to Duhem’s claims about blaming auxiliary assumptions in efforts to “save” research.

There are also practical reasons to be skeptical of explaining all failures to replicate in terms of difference makers. There are potentially unlimited differences between two studies’ components, which might not explain a mismatch in their outcomes, and there is only so much time and so many resources researchers can devote to pursuing these differences while neglecting other epistemic aims. Researchers may be better off accepting that a failure of a reasonable effort to replicate shows that an original study is unreliable given its scope, after which they can change their methods or address other claims that might fulfill their full set of epistemic aims.

Our claims are consonant with those of philosophers and scientists who argue that pursuing replication should not lead us to overlook other epistemic aims, such as validity (Feest 2019) or generalizability (Yarkoni 2019). However, we do not challenge the importance of replication. By contrast, we recognize its importance and the corresponding importance of how researchers respond to reported replication failures. Reliability is just one of several epistemic aims, but it is important. Mismatch explanations can be defensible. However, citing differences without consideration of relevance can undercut other epistemic aims. This is the case even if looking for difference makers seems, at first glance, appropriate. The concern that researchers will focus on difference makers at the expense of representativeness and scope of studies is pronounced in neuroscience, where translation is often a long-term epistemic aim, organisms are frequently deployed, and a multiplicity of experimental protocols (Sullivan 2009) make salient the myriad ways in which two studies can differ.

### Our recommendation

For a positive conclusion, we propose a method for how researchers should approach replication and figure out whether a mismatch explanation is appropriate. Before a replication is attempted, replicators should present an experimental protocol to the original researchers and ask these researchers if the protocol matches the original study. Original researchers can check whether fixed factors are matched and whether random factors are representative of the population of the original study’s random factors. A discussion should then be given to factors identified by the original researchers as having to be altered to match appropriately, and particular attention should be given to the extent to which these factors are allowed to vary and how such freedom or constraint might affect the scope and other epistemic aims of the research.

Our proposal capitalizes on the fact that the difference maker and relevance conditions are independent of one another. Because a difference can be a difference maker without satisfying the relevance condition and vice versa, researchers can resolve whether the relevance condition is met before any difference makers are found. Doing this first prevents the possibility that researchers will explain a possible failure to replicate in terms of a difference maker simply because this difference maker has been discovered or even merely speculated upon. It also prevents researchers needlessly spending time, money, and resources hunting down potential difference makers that are irrelevant to explaining reported replication failures.

Our proposal resolves the potential issue that researchers can be vague about the exact scope of the original study’s outcomes and its components’ representativeness. Researchers might find it difficult to express these commitments a priori and in the abstract, but this proposal requires them only to identify whether a particular component is representative of the appropriate population. By comparing a particular experimental protocol to another protocol, the exact scope and representativeness of the original study need not be precisely stated.

Our proposed method would not stop mismatches from occurring. Researchers still might inadvertently introduce non-matching components into their studies. However, our proposal supplies an answer to how difference makers should be evaluated if, or perhaps even before, they are discovered. If these differences do not fulfill the relevance condition and this fact is discussed ahead of time, they cannot be invoked as part of a mismatch explanation. And, if difference makers are discovered, researchers still can investigate them. Nothing about our recommendation, and nothing about our concerns with inappropriate appeals to mismatch explanations more generally, suggests that researchers should not pursue difference makers as a means of generating scientific knowledge. However, they should investigate them because they are independently interesting, not because they explain a reported replication failure.

## Conclusion

Though mismatch explanations might seem like the scapegoating of the inevitable differences between studies, there are cases where these explanations are appropriate. However, they are not always appropriate, and citing differences can undermine other epistemic aims. Given the generality with which scientists often present their outcomes, we should take seriously these potential negative consequences when citing study differences. If an epistemic aim like translation is the end goal, as it was in FORE-SCI, then citing irrelevant differences, even when they are difference makers, may move scientists farther from this goal.

While we stress the potential negative consequences of citing study differences, we do not fault the individual scientists who do this. We speculate that scientific disciplines favor a focus on differences for at least three reasons. First, as noted above, sometimes seeking out and correcting component differences is defensible. There are special cases when these may be the most salient aspect to pursue, such as when an experimental method involves a new technique or technology, making it more likely that the methods are not fully disseminated in detail or that replicating labs may take some time to perfect their use of a new technique. Likewise, difference makers can be worth pursuing for scientific aims that are independent of reliability or replication. For this reason, it is perhaps not surprising that researchers might erroneously equate an interesting difference maker with one relevant to diagnosing a replication attempt.

Second, the endeavor to replicate another’s work may be viewed by researchers in terms of a “prove or disprove” mentality. Despite attempts to portray replication in a neutral, blameless manner, the goal after a failure to replicate may be less “is this outcome reliable?” and more “how did the original team mess up (or lie)?” Citing differences supplies a means of placing the error in the precision of the replication, rather than blaming the original researchers for incompetent or immoral research practices. Indeed, the penalties for dishonesty are harsh (and one could certainly argue that they should be), but even a whiff of dishonesty can ruin careers. Thus, there may be reluctance among anyone suspicious of an original study to become an accuser of scientific incompetence or fraud.

Third, placement of the error on differences also alleviates the entire field of researchers from blame for systemic practices, such as publication bias or undue focus on novelty in grant funding, which may contribute to failures of replication or even a lack of attempts at replication. This might explain why programs like FORE-SCI are uncommon, as funding often is not provided for research focused solely on replication, and why failures to replicate often go unpublished. If these speculations are correct, they suggest that there may be a need for us to consider the importance or value of replication as it relates to replication failures.

## Footnotes

1 This is not to say that original researchers’ intentions are necessarily the best way in which the study in question could be performed. What matters in replication, and what distinguishes it from a mere follow-up, is that the replication attempt fits the fixed and random factors of the original study.

2 This need not be inadvertent. For instance, replicators might not include a component if the original report does not state it explicitly (Hensel 2020).

3 Our account thus supplies a more precise basis to assess mismatch explanations than stating that “critical features” must be duplicated in a replication (Zwaan et al. 2018, p. 6; Romero 2019).

4 As Zwaan and colleagues note, “if a failed replication brings to light some factor that could potentially affect the result and that differed between the original study and the replication… the post hoc consideration of differences in features should lead to new testable hypotheses rather than blanket dismissals of the replication result” (2018, p. 6).

5 We thank an anonymous reviewer for raising this question.
